# Prospective Acid Reflux Study of Iran (PARSI): Methodology and study design

**DOI:** 10.1186/1471-230X-7-42

**Published:** 2007-11-20

**Authors:** Siavosh Nasseri-Moghaddam, Hadi Razjouyan, Seyed Maysam Alimohamadi, Mansoureh Mamarabadi, Mohamad-Hamed Ghotbi, Pardis Mostajabi, Amir-Ali Sohrabpour, Masoud Sotoudeh, Behnoush Abedi, Azadeh Mofid, Mehdi Nouraie, Shahnaz Tofangchiha, Reza Malekzadeh

**Affiliations:** 1 Digestive Disease Research Center (DDRC), Shariati Hospital, Medical Sciences/University of Tehran, North Kargar Ave., Tehran, Iran

## Abstract

**Background:**

Gastroesophageal reflux disease is a common and chronic disorder but long term, prospective studies of the fate of patients seeking medical advice are scarce. This is especially prominent when looking at non-erosive reflux disease (NERD) patients.

**Methods:**

We designed a prospective cohort to assess the long term outcome of GERD patients referring to gastroenterologists. Consecutive consenting patients, 15 years of age and older, presenting with symptoms suggestive of GERD referring to our outpatient clinics undergo a 30 minute interview. Upper gastrointestinal endoscopy is performed for them with protocol biopsies and blood samples are drawn. Patients are then treated according to a set protocol and followed regularly either in person or by telephone for at least 10 years.

**Discussion:**

Our data show that such a study is feasible and follow-ups, which are the main concern, can be done in a fairly reliable way to collect data. The results of this study will help to clarify the course of various subgroups of GERD patients after coming to medical attention and their response to treatment considering different variables. In addition, the basic symptoms and biological database will fuel further molecular epidemiologic studies.

## Background

Gastro-esophageal reflux disease (GERD) is a common and chronic problem [1]. The west has faced a dramatical increment in the incidence and prevalence of the disease over the past couple of decades [2,3]. Recent reports from developing countries indicate a similar trend [3-5]. In addition to imposing a large financial burden on the health care system, GERD affects different aspects of the patients' health including their quality of lives [6,7]. Therefore, it has received extensive attention during the past two decades. Conventionally, endoscopy is used to make the diagnosis, look for complications (e.g. Barrett's esophagus, strictures, ulcers, and adenocarcinoma) and rule out concomitant benign or malignant diseases [8]. Finding endoscopic esophagitis secures the diagnosis and its healing is commonly used as an endpoint for successful treatment. In a recent endoscopic survey in Iran, about 37% of the population had endoscopic esophagitis [9].

GERD patients are a heterogeneous group. They may be categorized considering their symptoms and endoscopic findings. From an endoscopic point of view, GERD is classified as those with no recognizable esophageal erosion (non erosive reflux disease, NERD), those with visible distal esophageal erosions (erosive reflux disease, ERD), and those with columnar metaplasia in the distal esophagus (Barrett's esophagus, BE). Occurrence of symptoms is not predictable by endoscopic findings, but it appears to be influenced by age, body mass index (BMI), alcohol, and cigarette use [[3,10], and [11]]. However, our knowledge about the differences in risk factors for NERD, ERD, and BE is very little. Neither typical symptoms nor amount of acid exposure of the esophageal mucosa can adequately predict the occurrence and severity of esophagitis [12-15]. It has been shown that long-lasting GERD symptoms can be a risk factor for BE [16,17]. Patients who develop BE may have an increased risk of esophageal adenocarcinoma. Adenocarcinoma has been reported in up to 10% of patients with BE at the time of the first upper endoscopy [18].

Large prospective studies will improve our understanding of the epidemiology, natural history, and complications of GERD [19]. There have been a few endoscopy-based cohorts with a follow-up period of more than twelve months [20-22]. Hereby we describe the methodology of our study aimed at following a cohort of 1,200 GERD patients for at least 10 years.

## Objectives

The primary objective of this ongoing study is to determine the response of GERD patients to medical therapy, factors influencing their response, assess interaction between various clinical and demographic features and the need for maintenance therapy. Secondary objectives include evaluation of (1) course of the disease in patients after consulting a physician, (2) factors affecting regression and relapse of GERD related symptoms, (3) the association between various risk factors and the progression of GERD and BE, (4) the socioeconomic costs of the disease, (5) the relationship between Helicobacter pylori infection and its eradication and GERD, (6) the interaction between genetic factors, serologic and histologic findings and the course of GERD.

Therefore, we developed a database of GERD patients to observe their overall outcomes and assess risk factors, diagnostic and therapeutic methods as they develop, and the health related quality of life of different subgroups described above before and after treatment. Other goals of the study are assessing the difference in severity of symptoms, responding to treatment, and the need for maintenance therapy in the following subgroups: patients with erosive reflux and non-erosive reflux disease (ERD vs. NERD), different grades of esophagitis, different presenting esophageal symptoms, and patients with esophageal and extra-esophageal symptoms. We also assess the difference in responding to medical treatment and the need for maintenance therapy between patients in whom helicobacter pylori (HP) has been eradicated for other indications and those who are still HP positive. In addition, the relation between cytochrome P-450 (e.g. CYP-450-IIC19) polymorphism and response to treatment and the need for maintenance therapy are assessed. We will also determine the frequency of other concomitant GI and non-GI disorders among the patients in the cohort especially that of irritable bowel syndrome (IBS), the metabolic syndrome (MES) and peptic ulcer disease (PUD).

## Methods/Design

### Study design

The Prospective Acid Reflux Study of Iran (PARSI) is a prospective study being conducted in Tehran, Iran in which patients with symptoms suggestive of GERD are followed prospectively in a set protocol for at least 10 years.

The patients are not paid for participation in the study. Any evaluation which is not part of the routine clinical care of these patients will be covered totally by the investigators and the Digestive Disease Research Center (DDRC) of Tehran University of Medical Sciences (TUMS). The study has been approved by the ethics committee of TUMS and is in accord with the declaration of Helsinki. All patients provide written informed consent before enrollment. Not giving an informed consent does not prevent the patient from receiving quality clinical care.

Educational sessions were provided for the investigators to assure standardization of the procedures, interviews and conducting the study.

### Patients

**Figure 1:**
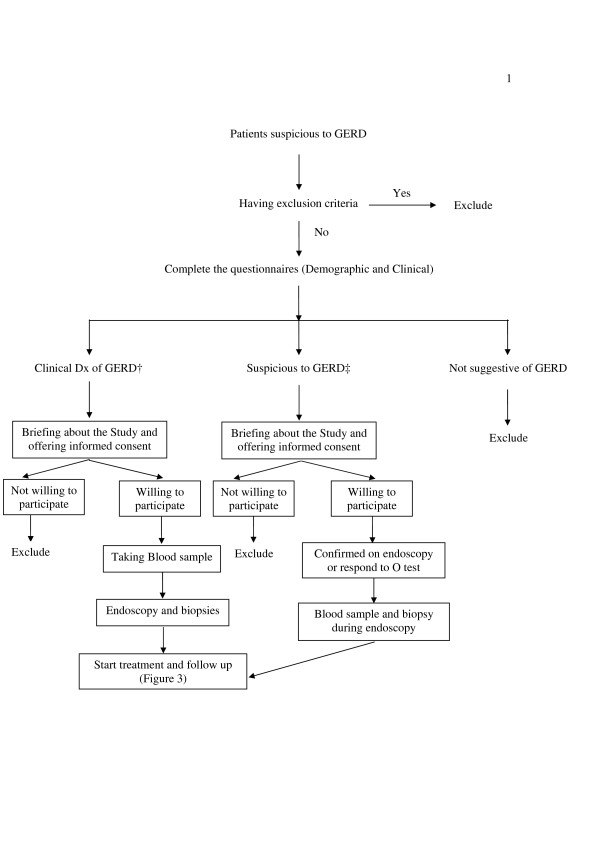
**Flow chart of patient enrolment**. †: Presence of at least one major symptom with appropriate duration, ‡: Presenting just by minor symptoms.

All consenting patients, 15 years of age and older, presenting with symptoms suggestive of GERD referring to our outpatient clinics (Behrouz and Iran-e-ma) undergo a 30 minute interview; this is done by a trained general practitioner. All patients who have major symptoms (Table 1) for at least 4 weeks over the past three months are enrolled. Those presenting by minor symptoms (Table 1) are eligible if they have a positive omeprazole test (subsidence of symptoms by omeprazole 20 mg orally twice daily for 4 weeks) or have erosions on endoscopy. Exclusion criteria are shown in Table 2. Study flow chart is shown in Figure 1.

**Table 1:** Major and minor symptoms of GERD

**Major**	**Minor**
HeartBurn (HB)	Chronic interscapular pain
Acid Regurgitation (AR)	Halitosis
Dysphagia (D)	Bitter mouth
Non Cardiac Chest Pain (NCCP)	Water brash
	Anorexia
	Nausea
	Hoarseness
	Globbus sensation
	Chronic cough
	Sore throat/mouth
	Belching

**Table 2:** Exclusion Criteria

Esophageal varices
Pregnancy
Advanced cardiovascular disease
History of upper GI surgery
Esophageal or gastric cancer
Using H2 blocker and/or PPIs during the last 2 weeks*

*: These patients were eligible if erosions were seen on endoscopy.

### Demographic and medical questionnaires

At the baseline visit, each participant's medical history including information on frequency, severity, and intensity of major and minor GERD symptoms, duration of the symptoms, chief complaint taking him/her to the physician, coffee, tea and alcohol consumption, and up to five specific foods which exacerbate their symptoms are recorded. Another questionnaire including demographic data (age, sex, place and date of birth, job, marital status, education, weight, height, income status), habits [exercise, smoking, opium use, the interval between having dinner to going to bed (dinner to bed time), sleep position, and alcohol intake], past medical history (any comorbid diseases or surgeries and type of delivery in females), family history (any problem with their upper GI tract in first degree family members and spouse), and drug history is also filled out. Average salt intake is estimated through dietary habit assessment.

### Blood samples

Twelve milliliters of venous blood is taken before upper GI endoscopy. Both EDTA and clot aliquots are prepared and kept in -70°C freezers for further analysis.

### Endoscopy and biopsies

**Figure 2:**
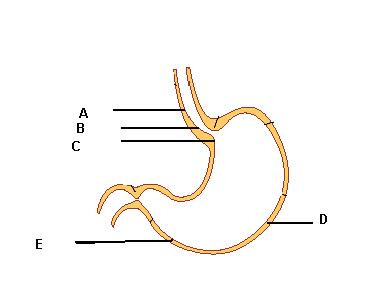
**Places of taking biopsies**. A: lower third of esophagus within 2 cm from the z-line, B: Gastroesophageal junction across the z-line, C: Cardia, D: Body, E: Antrum.

Upper GI endoscopy is performed in a left lateral position with local anesthesia ± intravenous midazolam according to patients' need and preference. Structural and mucosal details of the esophagus, stomach, and duodenum down to its second portion are recorded. Esophagitis is recorded using the Los Angeles classification [23]. Routine antral biopsy for rapid urease test (RUT) is taken and all lesions needing biopsy, as judged by the endoscopist, are biopsied. Thereafter, protocol biopsies (Figure 2) are taken.

Biopsies are fixed in 10% buffered formalin; standard 4 micrometer thick sections are prepared and stained with hematoxilin and eosin (H & E). The Sydney system is used for interpretation of gastric biopsies [24].

### Treatment protocol

**Figure 3:**
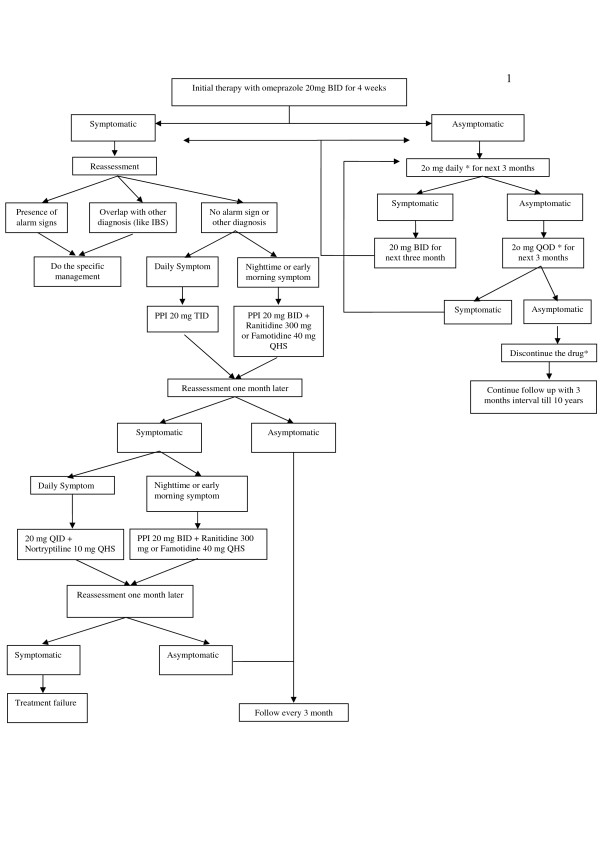
**Therapy flow chart of the study**. * Whenever the dosage is tailored, patients should assess her/his well being after one week. If patient is better with the previous prescription, patient should back to the previous dosage and continue till the next visit.

A step down protocol is used for treatment. Omeprazole 20 mg twice daily (half an hour before breakfast and dinner) is given for the initial four weeks. The patients are reassessed and checked for compliance, symptoms, and adverse events initially at week 4. The dosage is tailored in a step down manner according to patient satisfaction and/or symptoms following a set protocol (Figure 3). Then, the patients are followed every twelve weeks either in person at the office or via telephone, until endpoints are achieved. Thereafter, the patients are followed as necessary or at least every 6 months for 10 years.

Helicobacter Pylori (HP), if present, is eradicated only if there is a clear indication for it (i.e. duodenal or gastric ulcer or erosions, family history of gastric cancer or patient's preference for HP to be eradicated).

During the initial healing phase, the study medication is the only acid-suppressing drug to be administered. Other medications considered necessary for the patient's safety and well-being can be used or continued.

### Follow up

At the base line visit, a pamphlet containing life style modifications considered appropriate for GERD is given to the patients in addition to detailed verbal explanation. Patients are followed at regular three month intervals. If patients do not show up for two weeks or more after a scheduled visit, they are contacted by phone. In each follow up, clinical questionnaire will be filled out again. Patient's weight and drug consumption including PPI and other drugs are asked. All patients are planned to be followed for 10 years.

### Outcomes and outcome measures

Quality of life is measured with the disease-specific "Quality of Life in Reflux and Dyspepsia" questionnaire (QOLRAD) that is specifically developed and validated to assess the impact of heartburn and has been validated in Iran [25]. Generic QoL is measured using the "Medical Outcome Study Short Form 36 (SF-36) Health Survey" which has also been validated in Iran [26].

Primary outcomes of interest include: Complete response (remission off treatment or remission with maintenance treatment), partial response, and non-response. At the initial visit a "General Symptom Score" (GSS) and a "Major Symptom Score" (MSS) is calculated for each patient using frequency, intensity, and severity of each symptom (Table 3 and 4). The GSS ranges from zero to 382. Patients' response to treatment is defined according to the general symptom score (GSS) and major symptom score (MSS) and self satisfaction. Accordingly, patients are categorized as complete, partial, or non-responders. In each follow up symptom score reduction ratio is calculated [Symptom Score Reduction Ratio (SSRR) = (Symptom score_n _– Symptom score_n-1 _)/Symptom score _n_, where "n" is the number of the last visit]. Complete responders are those whose major symptom resolves or have occasional symptoms (less than one episode per week) or have a general and major SSRR ≥ 75%. Partial responders are those who have ≥ one episode of their major symptom per week and are sub grouped to well and fair groups. Well patients are those who had general or major SSRR of 50%–74%. General or major SSRR of Fair subgroup is 25%–49%. Those who have a less than 25% SSRR are considered as non-responders. If the patient's medication can not be decreased on 2 successive visits, he/she is considered to need long-term maintenance. Anti-reflux surgery is offered to this subgroup. If symptoms recur at any time after discontinuation of medications, the patient is re-assessed clinically, possible risk factor(s) identified and treatment restarted accordingly.

**Table 3:** Questions and scores used for evaluating frequency, intensity, and severity of GERD related symptoms

**Symptoms**	**Frequency**					**Intensity**	**Severity**		
Heartburn, Acid regurgitation, Non Cardiac Chest Pain, Chronic Interscapular Pain	None (0)	With specific food (1)	Less than weekly (2)	1–3 times per week (3)	Almost every day (4)	Several times a day (2×)	Do not feel it if don't think about it (2)	Feel it, but not interfering with work and/or daily life (3)	Interfering with work and/or daily life (4)
Halitosis, Water Brash, Nausea, Hoarseness, Globus, Bitter Mouth	None (0)		Less than weekly (1)	1–3 times per week (2)	Almost every day (3)	Several times a day (2×)	Do not feel it if don't think about it (2)	Feel it, but not interfering with work and/or daily life (3)	Interfering with work and/or daily life (4)
Dysphagia, Odynophagia	None (0)	With specific food or condition (1)	Less than weekly (2)	1–3 times per week (3)	Almost every day (4)		Relieves spontaneously (2)	Relieved with drinking water(3)	Only partially relieved by ingesting large amounts of water (4)
Chronic Cough, Sore throat and mouth	None (0)		Occasionally (1)	Most days (2)	Almost every day (3)	Almost all day long (2×)	Not interfering with work and/or daily life (2)	Interfering with work and/or daily life (3)	
Anorexia	None (0)		Less than weekly(1)	1–3 times per week (2)	Almost every day (3)	Several times a day (2×)	Although present but I take almost normal amount of food (2)	I have cut my food intake (3)	I am almost unable to take any food (4)
Belching	None (0)	With specific food or condition (1)	Less than weekly (2)	1–3 times per week (3)	Almost every day (4)	Almost all day long (2×)	Not interfering with work and/or daily life (2)	Interfering with work and/or daily life (3)	

**Table 4:** An example of calculating the symptom score. For a 45 years old gentleman presenting with the following symptoms for the past 5 months. The Symptom Scores will be calculated as follows:

**Symptoms**	**Frequency**					**Intensity**	**Severity**		
Heartburn	None (0)	With specific food (1)	Less than weekly (2)	**1–3 times per week (3)**	Almost every day (4)	**Several times a day (2×)**	Do not feel it if don't think about it (2)	**Feel it, but not interfering with work and/or daily life (3)**	Interfering with work and/or daily life (4)
Acid regurgitation	None (0)	With specific food (1)	**less than weekly (2)**	1–3 times per week (3)	Almost every day (4)	Several times a day (2×)	**Do not feel it if don't think about it (2)**	Feel it, but not interfering with work and/or daily life (3)	Interfering with work and/or daily life (4)
Halitosis,	None (0)		Less than weekly (1)	1–3 times per week (2)	**Almost every day (3)**	**Several times a day (2×)**	Do not feel it if don't think about it (2)	**Feel it, but not interfering with work and/or daily life (3)**	Interfering with work and/or daily life (4)
Anorexia	None (0)		Less than weekly (1)	1–3 times per week (2)	**Almost every day (3)**	Several times a day (2×)	Although present but I take almost normal amount of food (2)	**I have cut my food intake (3)**	I am almost unable to take any food (4)
Belching	None (0)	With specific food or condition (1)	Less than weekly (2)	1–3 times per week (3)	**Almost every day (4)**	**Almost all day long (2×)**	**Not interfering with work and/or daily life (2)**	Interfering with work and/or daily life (3)	

Heartburn = 3*2*3 = 18

Acid regurgitation = 2*2 = 4

Halitosis = 3*2*3 = 18

Anorexia = 3*3 = 9

Belching = 4*2*2 = 16

General Symptom Score (GSS) = Heartburn + Acid regurgitation + Halitosis + Anorexia + Belching = 18+4+18+9 +16 = 64

### Data management and statistical analysis

During the follow-up phase all case report forms will be manually checked for completeness and relevant missing data will be corrected by additional telephone questioning. Data are then transmitted to a data base specifically designed for this study. The data base is maintained in the central computer of the GERD study group at the digestive disease research center of Tehran University of medical sciences. Statistical calculations are performed using SPSS 16.0 (SPSS Inc., Chicago, IL, USA) and STATA 9.0 (Stata corp., College Station, Texas). Various statistical tests are used according to need.

## Discussion

We describe the protocol of a prospective cohort study in patients presenting with GERD related symptoms. This study will provide important new information on a highly controversial area of characteristics of different subgroups of GERD patients and their response to treatment. Though it is not a randomized trial, we believe a carefully designed and analyzed cohort study will improve our knowledge of both treatment efficacy and safety in routine practice. The primary aim of this study is to better determine the outcomes of GERD patients as it is practiced today and will provide useful information on long term follow-up of these patients. The results may help improve the selection criteria for medical therapy and even surgery, better define GERD related symptoms and its spectrum, the prognosis following therapy, and improve our ability to predict patients with the most favorable treatment approaches.

Our data show that such a study is feasible and follow-ups, which are the main concern, can be done at a fairly reliable way. The results of this study will help to clarify the course of various subgroups of GERD patients after coming to medical attention and their response to treatment considering different variables. In addition, the basic symptoms and biological database will fuel further molecular epidemiologic studies.

## Abbreviations

**NERD**: Non-Erosive Reflux Disease; **ERD**: Erosive Reflux Disease; **BE**: Barrett Esophagus; **GERD**: Gastro-Esophageal Reflux Disease; **IBS**: Irritable Bowel Syndrome: **MES**: Metabolic Syndrome; **PUD**: Peptic Ulcer Disease; **PARSI**: Prospective Acid Reflux Study of Iran; **DDRC**: Digestive Disease Research Center; **TUMS**: Tehran University of Medical Sciences; **RUT**: Rapid Urease Test; **GI**: Gastro-Intestinal; **HP**: Helicobacter Pylori; **QOLRAD**: Quality of Life in Reflux and Dyspepsia; **GSS**: General Symptom Score; **MSS**: Major Symptom Score; **SSRR**: Symptom Score Reduction Ratio.

## Competing interests

The author(s) declare that they have no competing interests.

## Authors' contributions

All authors participated in conception, design and acquisition of data. RM and SN-M did all upper GI endoscopies and Biopsies. MS, and BA were the pathologist. S N-M, H R, M M, A M, M N, R M, and SM A contribute in analysis and interpretation of data and revising for important intellectual content. Final approval of the manuscript for publishing was done by all the authors.

## Pre-publication history

The pre-publication history for this paper can be accessed here:


